# Odontogenic myxoma of the maxilla invading the maxillary sinus

**DOI:** 10.1016/S1808-8694(15)30159-2

**Published:** 2015-10-18

**Authors:** Ruy de Oliveira Veras Filho, Silvano Santos Pinheiro, Isabel Cristina Pinheiro de Almeida, Maria de Loudes Silva Arruda, Antonio de Lisboa Lopes Costa

**Affiliations:** a Graduate student in Dentistry and Maxillo-Facial Surgery - UFPel; b Specialist in Maxillo-Facial Surgery - UFPel and Master”s Degree Student in Stomatology - UFPB, Professor of Oral Oncology - Hospital Dr. Luiz Antônio da LIGA Norte-Rio-Grandense Contra o Câncer; c Head and Neck Surgeon - hospital Dr. Luiz Antônio da LIGA Norte-Rio-Grandense Contra o Câncer; d MSc. In Oral Pathology - UFRN e PhD Student in Health Sciences - UFRN, Oral oncology Program Coordinator and in charge of the Department of Dentistry - hospital Dr. Luiz Antônio da LIGA Norte-Rio-Grandense Contra o Câncer; e MSc. In Oral Pathology - UFRN PhD in Oral Pathology - FO-USP, Adjunct Professor of Oral Pathology - UFRN, Professor of the Master”s and PhD Programs in Oral Pathology - UFRN and head of the Department of Dentistry - UFRN; f Liga Norte-Rio-Grandense Contra o Câncer-Hospital Dr. Luiz Antônio

## INTRODUCTION

Dental tumors are lesions that stem from the epithelial and mesenchymal elements which are tooth components. For this very reason, they involve the maxilla and must make up the differential diagnosis of the lesions associated with this structure. The odontogenic myxoma is an uncommon benign neoplasia of slow and infiltrative growth, usually assymptomatic^1^. It may happen at any age; however, it is more common in the second and third decades of life. The mandible is more affected than the maxilla, and the posterior mandible is the region with the highest occurrence. Radiographically speaking, the myxoma is radiolucent, it can present itself as well outlined or diffuse, uni or multilocular and it can shift or cause tooth resorption in the tumor area. The differential diagnosis includes cysts, ameloblastomas, gigantic cell central granulomas, fibroma and fibrous dysplasia^1^, ^4^. Treatment of choice is surgical resection, and it recurs at a significant rate of about 25%; there are no metastases and prognosis is favorable^1^, ^2^. Proservation must be strict and patient rehabilitation is paramount, since the treatment itself is mutilating.

## CASE PRESENTATION

**Figure 1:**
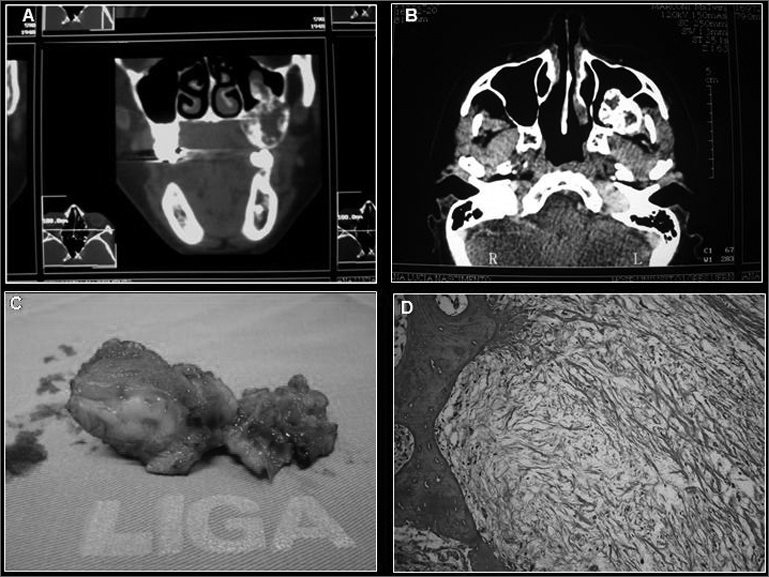
A- CT scan coronal section, B- CT scan axial section, C-Surgical Specimen, D- Histopathology Cross-Section.

ALNM, a 38-year-old female, African-American, came to de dentistry department of the hospital with a posterior mass in her left maxilla, without knowing for certain the time of evolution, and reporting that she had been submitted to surgery to correct the alveolar border with a histopathology diagnosis of myxofibroma. Panoramic x-ray showed an imaging suggesting a multilocular bone destruction of ill-defined margins. A CT-scan showed involvement of the left maxillary sinus, with evidence of lesion infiltration (Figs. 1A and 1B). The pathology report on the incisional biopsy was of odontogenic myxoma, hereby described: “The cross-sections showed a para-keratinized pavimentous epithelium. The adjacent connective tissue showed areas of intensely myxoid material, with starshaped and spindle-like cells, and bundles of undulated connective tissue”, without the need for immunohistochemical tests to confirm the diagnosis. Treatment of choice was total lesion resection, partial mesio and infra-structure maxillectomy with bone margin and, for the procedure we used the Weber-Ferguson incision, thus providing a better access to the lesion in the maxillary sinus. The surgical specimen was referred to analysis and the diagnosis was settled in myxoma, repeating the histopathological findings (Figs. 1C and 1D). The patient has been followed up on a monthly basis for two years now, without evidence of lesion recurrence so far.

## DISCUSSION

In the aforementioned case, the patient came to us complaining of an asymptomatic growth in the oral region of the maxilla, which is in accordance with most of the authors who state that this is a slow growth, invasive, asymptomatic lesion with bone cortical expansion. The mandible is the most involved region^3^. Maxilla involvement is more frequent in the molar and zygoma region. In this case, the tumor was located in the left posterior maxilla region, invading the maxillary sinus. As far as the x-ray is concerned, most authors report a multilocular radiolucent image as the most commonly found - as it happened in our case, and in this image we did not see bone perforation and maxillary sinus invasion, which was proven by the CT scan, thus proving its importance for the treatment of the lesion^6^. The treatment performed was tumor resection with healthy tissue margins, which is the one most advocated by the authors, especially in the maxilla and the posterior region, because of the very difficulty in clinically establishing tumor invasion level and complete lesion removal by means of a more conservative technique4. Proservation is of two years, when most recurrences appear^1^, ^5^.

## FINAL REMARKS

Odontogenic myxoma is a rare lesion, with characteristics that are similar to those of other oral lesions, and this requires knowledge on the lesion, a detailed exam and histopathology in order to establish an early diagnosis and to offer a safe and risk-free treatment to the patient, avoiding sequelae and extensive mutilations which are common in the treatment of invasive lesions.
